# Entropy analysis of nickel(II) porphyrins network via curve fitting techniques

**DOI:** 10.1038/s41598-023-44000-1

**Published:** 2023-10-12

**Authors:** Muhammad Talha Farooq, Thiradet Jiarasuksakun, Pawaton Kaemawichanurat

**Affiliations:** 1 Department of Mathematics, Faculty of Science, King Mongkut’s University of Technology Thonburi, Bangkok, Thailand; 2 Mathematics and Statistics with Applications (MaSA), Bangkok, 10400 Thailand; 3 The Institute for the Promotion of Teaching Science and Technology (IPST), Bangkok, Thailand

**Keywords:** Cheminformatics, Physical chemistry

## Abstract

Nickel(II) porphyrins typically adopt a square planar coordination geometry, with the nickel atom located at the center of the porphyrin ring and the coordinating atoms arranged in a square plane. The additional atoms or groups coordinated to the nickel atom in nickel(II) porphyrins are called ligands. Porphyrins have been investigated as potential agents for imaging and treating cancer due to their ability to selectively bind to tumor cells and be used as sensors for a variety of analytes. Nickel(II) porphyrins are relatively stable compounds, with high thermal and chemical stability. They can be stored in a solid state or in solution without significant degradation. In this study, we compute several connectivity indices, such as general Randi’c, hyper Zagreb, and redefined Zagreb indices, based on the degrees of vertices of the chemical graph of nickel porphyrins. Then, we compute the entropy and heat of formation NiP production, among other physical parameters. Using MATLAB, we fit curves between various indices and the thermodynamic properties parameters, notably the heat of formation and entropy, using various linearity- and non-linearity-based approaches. The method’s effectiveness is evaluated using $R^2$, the sum of squared errors, and root mean square error. We also provide visual representations of these indexes. These mathematical frameworks might offer a mechanism to investigate the thermodynamical characteristics of NiP’s chemical structure under various circumstances, which will help us understand the connection between system dimensions and these metrics.

## Introduction

Transition metal (TM) porphyrins are widely used in a variety of technological applications, including sensors, pigment applications, cancer therapy, synthetic photosynthesis, nonlinear optics, and nanomaterials, as a result of their special features^1^. Their value for catalysis and biological significance is directly tied to this interest. The coordination characteristics and conformational flexibility of porphyrins have been extensively used over the past ten years in the quest for potential porphyrin isomers that can provide enhanced functionality in particular technological applications^2^. The nitrogen-confused porphyrins (NCPs), a unique and promising class of porphyrins with enhanced capabilities for application as acid catalysts and anion/cation sensors, are one such significant class of porphyrins^3^. The chemical structure, physical characteristics, and coordination properties of these porphyrin isomers are significantly different from those of the parent porphyrins. Such structures are great candidates for use in photodynamic therapy because of their effective singlet-oxygen sensitization^4^.

The information content of complex networks^5^ and graphs based on Shannon’s entropy^6^ work was first studied by researchers in the late 1990s. In discrete mathematics, computer science, information theory, statistics, chemistry, biology, and other domains, a large range of quantitative methods for studying complex networks have been developed^7,8^. For instance, graph entropy measurements have been extensively employed in the fields of mathematical chemistry, biology, and computer science to characterize the structure of graph-based systems^9,10^. To measure the structural complexity of graphs, the idea of graph entropy, created by Rashevsky^11^ and Trucco, has been employed^12^. Chemical indices are valuable resources for researching various physico-chemical characteristics of molecules without having to perform several tests. Quantitative structure-activity relationships (QSAR) are used in the study of drugs to understand the chemical properties using mathematical calculations^13,14^. The entropy of a graph was first described as an information-theoretic property by Mowshowitz^15^. Here, the complexity is clear. As stated by Shannon, uncertainty and information are two sides of the coin: a reduction in uncertainty is the same as the reception of a certain amount of information. Distinguished researchers have developed numerous techniques for efficiently computing structural descriptors, aimed at optimizing computational efficiency. Among these techniques, the polynomial representation of structural descriptors has garnered significant attention and widespread acceptance in the scientific literature^16,17^. Entropy has emerged as a comprehensive and overarching concept across a wide spectrum of disciplines, spanning from logic and biology to physics and engineering. It serves as the link between the ideas of randomness and uncertainty, connecting them with physical processes that are viewed as channels for the transformation of information^18^.

The visible-light-induced photo redox catalyst nickel(II) tetraphenyl porphyrin (NiTPP) is given as a reliable, affordable, and effective catalyst. Recently, it was demonstrated that a library of Ni(II) ligand-to-ligand charge transfer complexes has useful features as photosensitizers, but their use in conventional photoredox catalysis is still unexplored^19,20^. Porphyrins have a wide range of applications in various fields, including: Porphyrins have been investigated as potential agents for imaging and treating cancer due to their ability to selectively bind to tumor cells. They are also used in photodynamic therapy (PDT), a treatment method that uses light-sensitive compounds to destroy cancer cells^21^. Porphyrins and their derivatives have been widely studied as catalysts in organic chemistry, with applications in hydrogenation, oxidation, and cycloaddition reactions. Porphyrins have been used as sensors for a variety of analytes, including metal ions, pH, and gases^22^. Porphyrins have been used for the detection and removal of heavy metal ions from contaminated water^23^. Porphyrins have been used as natural pigments in plant breeding and as a growth regulator in crops^24^. Porphyrins have applications in biotechnology such as biosensors, bioimaging, and biocatalysis^25^.

Several well-known topological indices, or the values that help characterize a structure’s topological properties after it has been replicated, are used to calculate a structure’s degree-based entropy. Zhdanov examined the chemical processes involving organic compounds using entropy values^26^. Chen^27^ first defined the entropy of an edge-weighted graph in 2014. The information entropy is defined as:

1 $$\begin{aligned} E_{\phi }(\eta )=\log (I)-\frac{1}{I}\sum _{i=1}F_i\phi (a_ib_i)\log \phi (a_ib_i) \end{aligned}$$

In Eq. (1) $\eta _{e}$ is the edge set, $\eta _{v}$ is the vertex set, and $\phi (ab)$ is the edge weight of the edge (ab) in $\eta$ and $\eta =NiP$ be a molecular graph of Nickel Porphyrin and logarithm to be presumed to be based 10.

**Table 1:** Vertex partition of the Ni(II) porphyrins.

$(d_u)$	Frequency	Set of vertices
$d_2$	$16mn+6m+6n$	2
$d_3$	20*mn*	3
$d_4$	*mn*	4

**Table 2:** Topological characteristics and the edge’s (*mn*) weight are shown together.

Topological indices	$\phi (mn)$
The general *Randi*$\acute{c}$ index $R_{\alpha }=1,-1,\frac{1}{2},\frac{-1}{2}$^28^	$(\phi (m)\times \phi (n))^{\alpha }$
The atom bound connectivity index *ABC*^29,30^	$\sqrt{\frac{\phi (m)\times \phi (n)-2}{\phi (m)\times \phi (n)}}$
The geometric arithmetic index *GA*^29,30^	$\frac{2\sqrt{\phi (m)\times \phi (n)}}{\phi (m)+\phi (n)}$
The first Zagreb index $M_1$^29,30^	$(\phi (m)+\phi (n)$
The second Zagreb index $M_2$^29^	$(\phi (m)\times \phi (n)$
The hyper Zagreb index *H*^31^	$(\phi (m)\times \phi (n))^{2}$
The forgotten index *F*^32^	$(\phi (m)^{2}+\phi (n)^{2})$
The augmented Zagreb index *AZI*^33^	$(\frac{\phi (m)\times \phi (n)}{\phi (m)+\phi (n)-2})^{3}$
The first redefined Zagreb index $ReZG_1$^34^	$\frac{\phi (m)+\phi (n)}{\phi (m)\times \phi (n)}$
The second redefined Zagreb index $ReZG_2$^34^	$\frac{\phi (m)\times \phi (n)}{\phi (m)+\phi (n)}$
The third redefined Zagreb index $ReZG_3$^34^	$(\phi (m)\times \phi (n))(\phi (m)+\phi (n))$

## Structure of nickel(II) porphyrins

Nickel(II) porphyrins are a class of coordination complexes composed of a central nickel atom coordinated to four nitrogen atoms of a porphyrin ring and two additional atoms or groups^35^. These compounds are known for their stability, strong absorption in the visible region of the electromagnetic spectrum, and potential applications in catalytic and biomedical fields. They have been studied as catalysts in a variety of organic reactions, such as hydrogenation, oxidation, and cycloaddition reactions. In addition, they have been investigated as potential agents for imaging and treating cancer due to their ability to selectively bind to tumor cells. The additional atoms or groups coordinated to the nickel atom in nickel(II) porphyrins are called ligands. The ligands can vary depending on the synthesis method and the specific compound. Common ligands include water, chloride, and various organic groups^36^. Nickel(II) porphyrins have strong absorption in the visible region of the electromagnetic spectrum, with the absorption maximum typically around 400 nm. This property is due to the porphyrin ring and is used in applications such as imaging and photodynamic therapy^37^. Nickel(II) porphyrins have a low-spin electron configuration and have no unpaired electrons, so they have no net magnetic moment. Porphyrins and similar tetrapyrrolic macrocycles are found abundantly in nature and serve vital roles across a diverse range of disciplines, spanning from medicine to materials science. These compounds, particularly their metal complexes known as metalloporphyrins, serve as essential active centers in numerous enzymes^38^.

Since Küsterover^39^ initially postulated the porphyrin macrocyclic structure a century ago, study in the area has increased significantly, leading to a massive body of literature that is still growing quickly. To give you an idea, the “Handbook of Porphyrin Science” series,^40^ which was started in 2010, currently consists of 44 volumes and 214 chapters. The use of X-ray crystallography (including synchrotron) and neutron crystallography to determine the crystal structures of porphyrins has greatly aided the development to date. Currently, the Cambridge Structural Database has far more than 4000 porphyrin crystal structures (CSD)^41^. The structure of the Ni-metallated version, which has an interlayer spacing of 3.347 and Ni that is coplanar with the macrocycle, is otherwise comparable to that of Porphyrins. Using nanoelectrodes, Yoon^42^ assessed the electrical conductivity of two varieties of porphyrin wires. One type included 48 Ni(II) porphyrin moieties in directly meso-meso-connected Ni(II) porphyrin arrays. Because of the orthogonal arrangement of these arrays, consecutive porphyrins are aligned along the chain at right angles to one another. By using X-ray diffraction and a combination of single-crystal and solution resonance Raman studies, the structure of nickel(II) [Ni(P)] has been identified. Both resonance Raman spectroscopy and X-ray diffraction are approaches that are effective for examining porphyrin structure.

## Methodology

**Figure 1:**
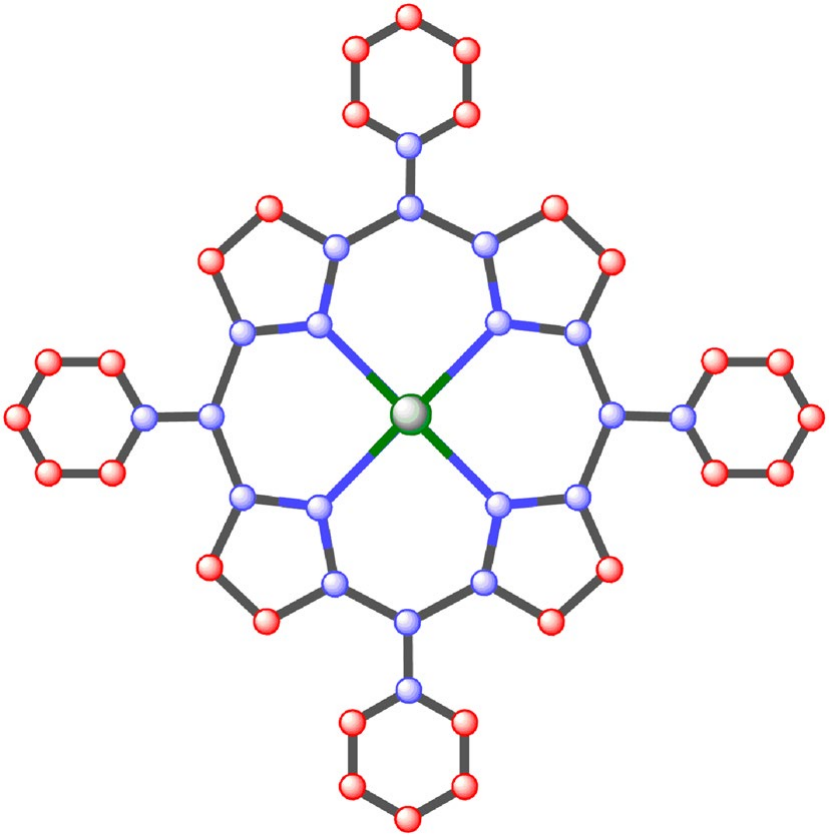
Unit structure of nickel(II) porphyrins [m = 1, n = 1].

**Figure 2:**
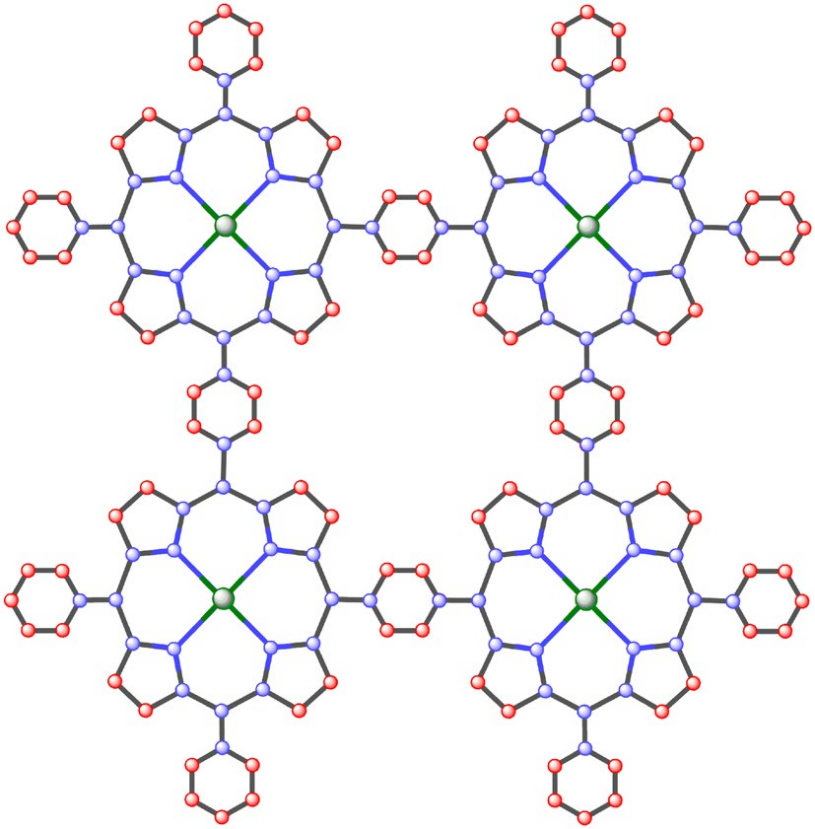
The structure of nickel(II) porphyrins [m = 2, n = 2].

**Figure 3:**
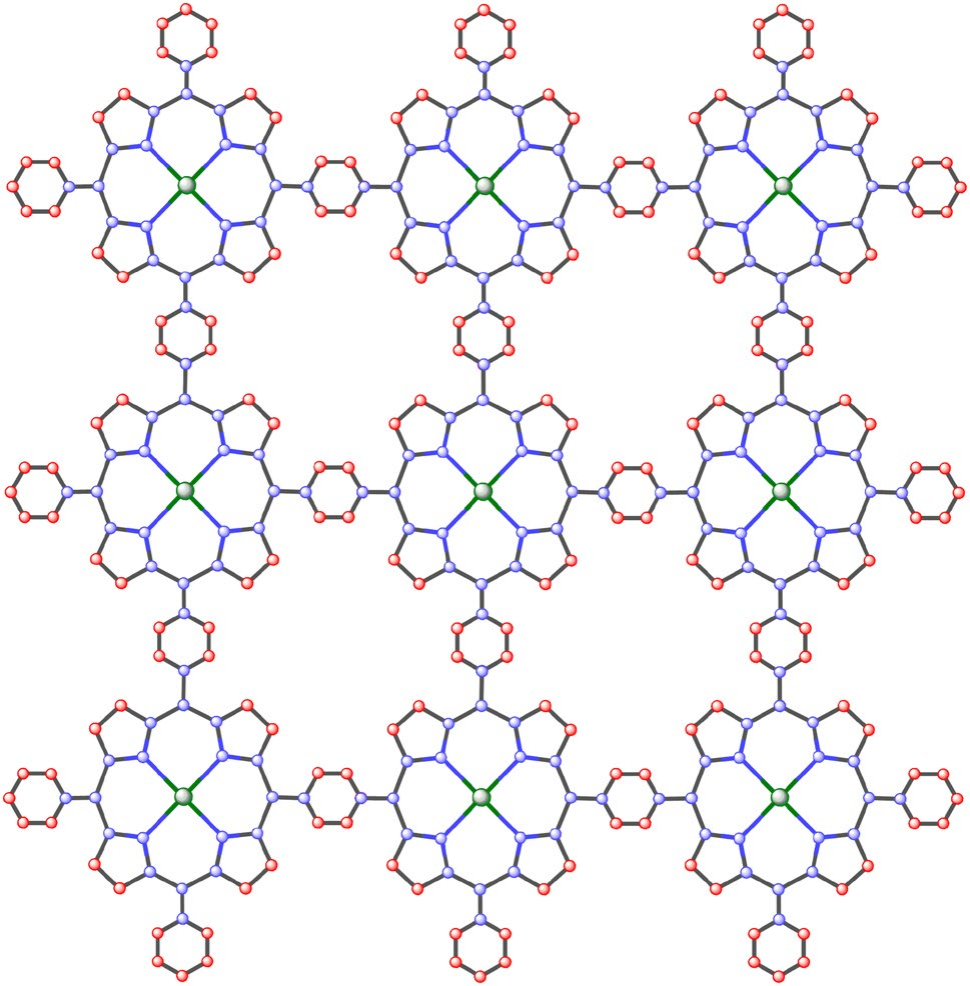
The structure of nickel(II) porphyrins [m = 3, n = 3].

Firstly, we find the degree of all types of vertices for the structure of nickel(II) porphyrins like we have three types of vertices: degree 2,3 and 4 and then we formulate general formulas for [*m*, *n*] dimensions and by utilizing these provided formulas in Table 1 we can compute vertices for any cell and by using same method we calculate the edge partition of nickel(II) porphyrins is shown in Table 3 and we have 4 types of edge partitions. The order and size of nickel(II) porphyrins for [*m*, *n*] is $37mn+6m+6n$ and $48mn+6m+6n$ respectively. Furthermore, degree-based topological indices that are mentioned in Table 2 are computed for $n\textrm{th}$ cell of nickel(II) porphyrins and entropy for these calculated indices by using Table 3 and explain it with numerical and graphical representation. And after that, we built-in function in MATLAB is used to create models between the Heat of Formation and each information entropy because it provides the lowest RMSE value, which indicates the best match. The Numerical Integrity of fit for entropy versus indices of Ni(II) porphyrins is depicted in Table 8. The unit structure of nickel(II) porphyrins (NiP) is shown in Fig. 1 and for more details about the structure of NiP(II) see the Figs. 2 and 3.

Using Table 3 and Equation the Randic^6^ and corresponding entropy for $\alpha =1,-1,\frac{1}{2},\frac{-1}{2}$ is :

**Table 3:** Edge partition of the Ni(II) porphyrins.

$(e_1,e_2)$	Frequency	Set of edges
(2, 2)	$8mn+6m+6m$	$E_1$
(2, 3)	16*mn*	$E_2$
(3, 3)	20*mn*	$E_3$
(3, 4)	4*mn*	$E_4$

## Computation of degree based indices and entropy of nickel(II) porphyrins [m,n]

### The Randi$\acute{c}$ index and Randi$\acute{c}$ entropy for Ni(II) porphyrins

Using Tables 2, 3 and Eq. (1) the Randic index^28^ and corresponding entropy is:

$$\begin{aligned} R_{\alpha }(NiP)=(8mn+6m+6n)\times (4)^{\alpha }+(16mn)\times (6)^{\alpha }+(20mn)\times (9)^{\alpha }+(4mn)\times (12)^{\alpha } \end{aligned}$$

$$\alpha =1$$

$$\begin{aligned} R_1(NiP)= & {} 356mn+24m+24n\\ E_{R_1}(NiP)= & {} \log [356mn+24m+24n]-\frac{(8mn+6m+6n) \log [4]^{4}}{(356mn+24m+24n)}-\frac{(16mn)\log [6]^{6}}{(356mn+24m+24n)}\\{} & {} - \frac{(20mn)\log [9]^{9}}{(356mn+24m+24n)}-\frac{(4mn)\log [12]^{12}}{(356mn+24m+24n)} \end{aligned}$$

$$\alpha = {-1}$$

$$\begin{aligned} R_{-1}(NiP)= & {} 7.2222222222mn+1.5m+1.5n\\ E_{R_{-1}}(NiP)= & {} \log [7.2222222222mn+1.5m+1.5n]-\frac{(8mn+6m+6n) \log \left[ (4)^{\left( \frac{-1}{4}\right) }\right] }{(7.2222222222mn+1.5m+1.5n)}\\{} & {} -\frac{(16mn)\log \left[ (6)^{\left( \frac{-1}{6}\right) }\right] }{(7.2222222222mn+1.5m+1.5n)}- \frac{(20mn)\log \left[ (9)^{\left( \frac{-1}{9}\right) }\right] }{(7.2222222222mn+1.5m+1.5n)}\\{} & {} -\frac{(4mn)\log \left[ (12)^{\left( \frac{-1}{12}\right) }\right] }{(7.2222222222mn+1.5m+1.5n)} \end{aligned}$$

$$\alpha = \frac{1}{2}$$

$$\begin{aligned} R_{\frac{1}{2}}(NiP)= & {} 129.0482424mn+12m+12n\\ E_{R_{\frac{1}{2}}}(NiP)= & {} \log \left[ 129.0482424mn+12m+12n\right] -\frac{(8mn+6m+6n)\log \left[ (4)^{\left( \frac{\sqrt{4}}{2}\right) }\right] }{(129.0482424mn+12m+12n)}\\{} & {} -\frac{(16mn)\log \left[ (6)^{\left( \frac{\sqrt{6}}{2}\right) }\right] }{(129.0482424mn+12m+12n)}- \frac{(20mn)\log \left[ (9)^{\left( \frac{\sqrt{9}}{2}\right) }\right] }{(129.0482424mn+12m+12n)}\\{} & {} -\frac{(4mn)\log \left[ (12)^{\left( \frac{\sqrt{12}}{2}\right) }\right] }{(129.0482424mn+12m+12n)} \end{aligned}$$

$$\alpha = \frac{-1}{2}$$

$$\begin{aligned} R_{\frac{-1}{2}}(NiP)= & {} 18.35333985mn+3.0m+3.0n\\ E_{R_{\frac{-1}{2}}}(NiP)= & {} \log [18.35333985mn+3.0m+3.0n] -\frac{(8mn+6m+6n)\log \left[ (4)^{\frac{-1}{2\sqrt{4}}}\right] }{(18.35333985mn+3.0m+3.0n)}\\{} & {} -\frac{(16mn)\log \left[ (6)^{\frac{-1}{2\sqrt{6}}}\right] }{(18.35333985mn+3.0m+3.0n)}- \frac{(20mn)\log \left[ (9)^{\frac{-1}{2\sqrt{9}}}\right] }{(18.35333985mn+3.0m+3.0n)}\\{} & {} -\frac{(4mn)\log \left[ (12)^{\frac{-1}{2\sqrt{12}}}\right] }{(18.35333985mn+3.0m+3.0n)} \end{aligned}$$

### The atom bond connectivity index and atom bond connectivity entropy for Ni(II) porphyrins

By using Tables 2, 3 and Eq. (1) the Atom bond connectivity index^29,30^ and corresponding entropy is:

$$\begin{aligned} ABC(NiP)= & {} 40.01062533mn+4.242640687m+4.242640687n \\ E_{ABC}(NiP)= & {} \log \left[ 40.01062533mn+4.242640687m+4.242640687n\right] \\{} & {} -\frac{(8mn+6m+6n)\log \left[ \left( \sqrt{\frac{4-2}{4}}\right) ^{\left( \sqrt{\frac{4-2}{4}}\right) }\right] }{40.01062533mn+4.242640687m+4.242640687n}\\{} & {} -\frac{(16mn)\log \left[ \left( \sqrt{\frac{6-2}{6}}\right) ^{\left( \sqrt{\frac{6-2}{6}}\right) }\right] }{40.01062533mn+4.242640687m+4.242640687n}\\{} & {} -\frac{(20mn)\log \left[ \left( \sqrt{\frac{9-2}{9}}\right) ^{\left( \sqrt{\frac{9-2}{9}}\right) }\right] }{40.01062533mn+4.242640687m+4.242640687n}\\{} & {} -\frac{(4mn)\log \left[ \left( \sqrt{\frac{12-2}{12}}\right) ^{\left( \sqrt{\frac{12-2}{12}}\right) }\right] }{40.01062533mn+4.242640687m+4.242640687n} \end{aligned}$$

### The geometric arithmetic index and geometric arithmetic entropy for Ni(II) porphyrins

By using Tables 2, 3 and Eq. (1) the Geometric Arithmetic index^29,30^ and corresponding entropy is:

$$\begin{aligned} GA(NiP)= & {} 36.70648944mn+6m+6n \\ E_{GA}(NiP)= & {} \log [36.70648944mn+6m+6n]-\frac{(8mn+6m+6n) \log \left[ \left( {\frac{2\sqrt{4}}{4}}\right) ^{ \left( \frac{2\sqrt{4}}{4}\right) }\right] }{36.70648944mn+6m+6n}\\{} & {} -\frac{(16mn)\log \left[ \left( {\frac{2\sqrt{6}}{6}}\right) ^{\left( \frac{2\sqrt{6}}{6}\right) }\right] }{36.70648944mn+6m+6n}-\frac{(20mn) \log \left[ \left( {\frac{2\sqrt{9}}{9}}\right) ^{\left( \frac{2\sqrt{9}}{9}\right) }\right] }{36.70648944mn+6m+6n}\\{} & {} -\frac{(4mn)\log \left[ \left( {\frac{2\sqrt{12}}{12}}\right) ^{\left( \frac{2\sqrt{12}}{12}\right) }\right] }{36.70648944mn+6m+6n} \end{aligned}$$

### The first Zagreb index and first Zagreb entropy for Ni(II) porphyrins

By using Tables 2, 3 and Eq. (1) the first Zagreb index^29,30^ and corresponding entropy is:

$$\begin{aligned} M_1(NiP)= & {} 260mn+24m+24n\\ E_{M_1}(NiP)= & {} \log [260mn+24m+24n]-\frac{(8mn+6m+6n)\log [4]^{4}}{(260mn+24m+24n)} -\frac{(16mn)\log [5]^{5}}{(260mn+24m+24n)}\\{} & {} - \frac{(20mn)\log [6]^{6}}{(356mn+24m+24n)}-\frac{(4mn)\log [7]^{7}}{(260mn+24m+24n)} \end{aligned}$$

### The second Zagreb index and second Zagreb entropy for Ni(II) porphyrins

By using Tables 2, 3 and Eq. (1) the second Zagreb index^29^ and corresponding entropy is:

$$\begin{aligned} M_2(NiP)= & {} 356mn+24m+24n\\ E_{M_2}(NiP)= & {} \log [356mn+24m+24n]-\frac{(8mn+6m+6n)\log [4]^{4}}{(356mn+24m+24n)}-\frac{(16mn)\log [6]^{6}}{(356mn+24m+24n)}\\{} & {} - \frac{(20mn)\log [9]^{9}}{(356mn+24m+24n)}-\frac{(4mn)\log [12]^{12}}{(356mn+24m+24n)} \end{aligned}$$

### The Hyper Zagreb index and Hyper Zagreb entropy for Ni(II) porphyrins

By using Tables 2, 3 and Eq. (1) the Hyper Zagreb index^31^ and corresponding entropy is:

$$\begin{aligned} HM(NiP)= & {} 1444mn+96m+96n \\ E_{HM}(NiP)= & {} \log [1444mn+96m+96n]-\frac{(8mn+6m+6n)\log [16]^{16}}{(1444mn+96m+96n)} -\frac{(16mn)\log [25]^{25}}{(1444mn+96m+96n)}\\{} & {} - \frac{(20mn)\log [36]^{36}}{(1444mn+96m+96n)} -\frac{(4mn)\log [49]^{49}}{(1444mn+96m+96n)} \end{aligned}$$

### The forgotten index and forgotten entropy for Ni(II) porphyrins

By using Tables 2, 3 and Eq. (1) the Forgotten index^32^ and corresponding entropy is:

$$\begin{aligned} F(NiP)= & {} 732mn+48m+48n\\ E_{F}(NiP)= & {} \log [732mn+48m+48n]-\frac{(8mn+6m+6n)\log [8]^{8}}{(732mn+48m+48n)} -\frac{(16mn)\log [13]^{13}}{(732mn+48m+48n)}\\{} & {} - \frac{(20mn)\log [18]^{18}}{(732mn+48m+48n)}-\frac{(4mn)\log [25]^{25}}{(732mn+48m+48n)} \end{aligned}$$

### The augmented Zagreb index and augmented Zagreb entropy for Ni(II) porphyrins

By using Tables 2, 3 and Eq. (1) the Augmented Zagreb index^33^ and corresponding entropy is:

$$\begin{aligned} AZI(NiP)= & {} 167.41928863mn+48m+48n\\ E_{AZI}(NiP)= & {} \log [167.41928863mn+48m+48n]-\frac{(8mn+6m+6n) \log [(\frac{64}{8})^{(\frac{64}{8})}]}{(167.41928863mn+48m+48n)}\\{} & {} -\frac{(16mn)\log [8]^{8}}{(167.41928863mn+48m+48n)}- \frac{(20mn)\log \left[ \left( \frac{729}{64}\right) ^{\left( \frac{729}{64}\right) }\right] }{(167.41928863mn+48m+48n)}\\{} & {} -\frac{(4mn)\log \left[ \left( \frac{1728}{125}\right) ^{\left( \frac{1728}{125}\right) }\right] }{(167.41928863mn+48m+48n)} \end{aligned}$$

### The first redefined Zagreb index and first redefined Zagreb entropy for Ni(II) porphyrins

By using Tables 2, 3 and Eq. (1) the first redefined Zagreb index^34^ and corresponding entropy is:

$$\begin{aligned} ReZG_1(NiP)= & {} 37mn+6m+6n\\ E_{ReZG_1}(NiP)= & {} \log [37mn+6m+6n]-\frac{(8mn+6m+6n) \log \left[ \left( \frac{4}{4}\right) ^{\left( \frac{4}{4}\right) }\right] }{(37mn+6m+6n)} -\frac{(16mn)\log \left[ \left( \frac{5}{6}\right) ^{\left( \frac{5}{6}\right) }\right] }{(37mn+6m+6n)}\\{} & {} - \frac{(20mn)\log \left[ \left( \frac{6}{9}\right) ^{\left( \frac{6}{9}\right) }\right] }{(37mn+6m+6n)} -\frac{(4mn)\log \left[ \left( \frac{7}{12}\right) ^{\left( \frac{7}{12}\right) }\right] }{(37mn+6m+6n)} \end{aligned}$$

### The second redefined Zagreb index and second redefined Zagreb entropy for Ni(II) porphyrins

By using Tables 2, 3 and Eq. (1) the second redefined Zagreb index^34^ and corresponding entropy is:

$$\begin{aligned} ReZG_2(NiP)= & {} 64.0571428571mn+6m+6n\\ E_{ReZG_2}(NiP)= & {} \log [64.0571428571mn+6m+6n] -\frac{(8mn+6m+6n)\log \left[ \left( \frac{4}{4}\right) ^{\left( \frac{4}{4}\right) }\right] }{(64.0571428571mn+6m+6n)}\\{} & {} -\frac{(16mn)\log \left[ \left( \frac{6}{5}\right) ^{\left( \frac{6}{5}\right) }\right] }{(64.0571428571mn+6m+6n)}- \frac{(20mn)\log \left[ \left( \frac{9}{6}\right) ^{\left( \frac{9}{6}\right) }\right] }{(64.0571428571mn+6m+6n)}\\{} & {} -\frac{(4mn)\log \left[ \left( \frac{12}{7}\right) ^{\left( \frac{12}{7}\right) }\right] }{(64.0571428571mn+6m+6n)} \end{aligned}$$

### The third redefined Zagreb index and third redefined Zagreb entropy for Ni(II) porphyrins

By using Tables 2, 3 and Eq. (1) the third redefined Zagreb index^34^ and corresponding entropy is:

$$\begin{aligned} ReZG_3(NiP)= & {} 2024mn+96m+96n\\ E_{ReZG_3}(NiP)= & {} \log [2024mn+96m+96n]-\frac{(8mn+6m+6n) \log [16]^{16}}{(2024mn+96m+96n)}-\frac{(16mn)\log [30]^{30}}{(2024mn+96m+96n)}\\{} & {} - \frac{(20mn)\log [54]^{54}}{(2024mn+96m+96n)}-\frac{(4mn) \log [84]^{84}}{(2024mn+96m+96n)} \end{aligned}$$

## Numerical and graphical representation of computed results

**Figure 4:**
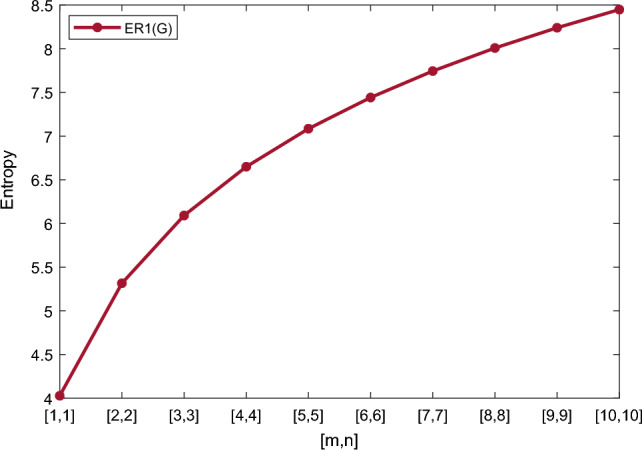
Graphically representation of $E_{R_1}$.

**Figure 5:**
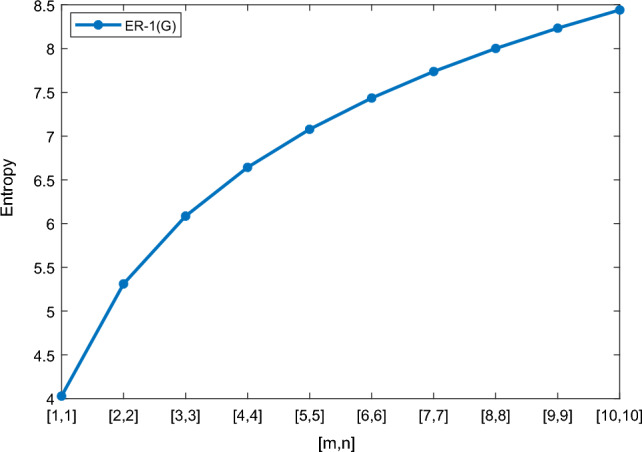
Graphically representation of $E_{R_{-1}}$.

**Figure 6:**
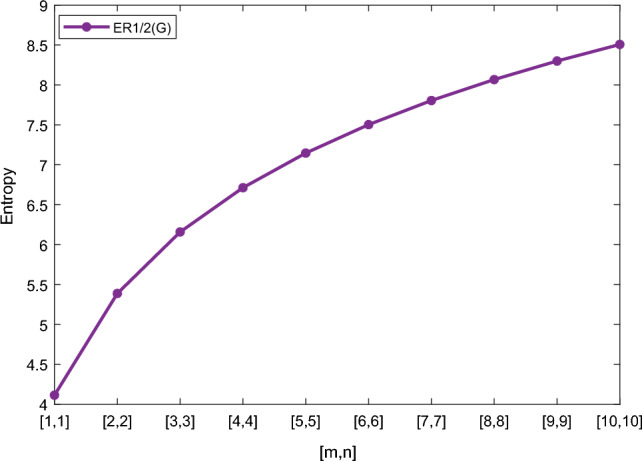
Graphically representation of $E_{R_{\frac{1}{2}}}$.

**Figure 7:**
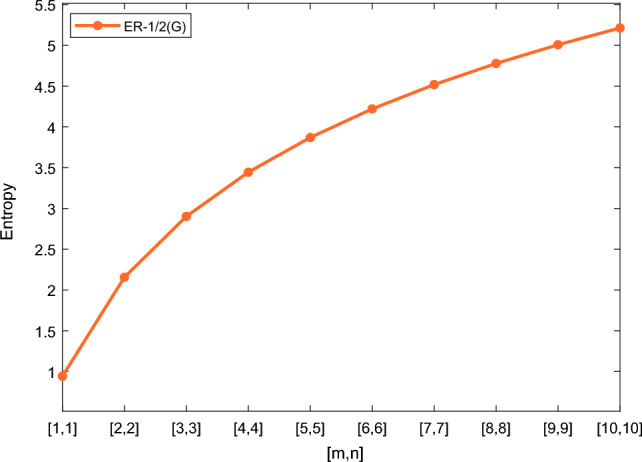
Graphically representation of $E_{R_{\frac{-1}{2}}}$.

In this section, we represent the numerical and graphical representation of the computed results. In Table 4 we represent the numerical results and in Figs. 4, 5, 6, 7 we represent the graphical comparison of the *Randi*$\acute{c}$ entropies for different values of $(\alpha =1,-1,\frac{1}{2}\, \,and\, \, \frac{-1}{2})$.

**Figure 8:**
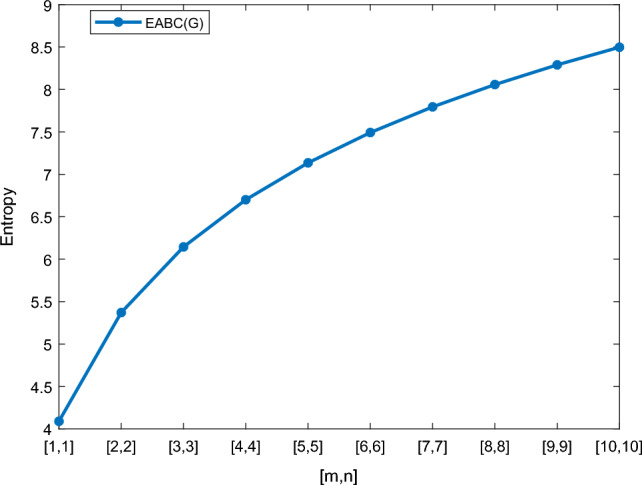
Graphically representation of $E_{ABC}$.

**Figure 9:**
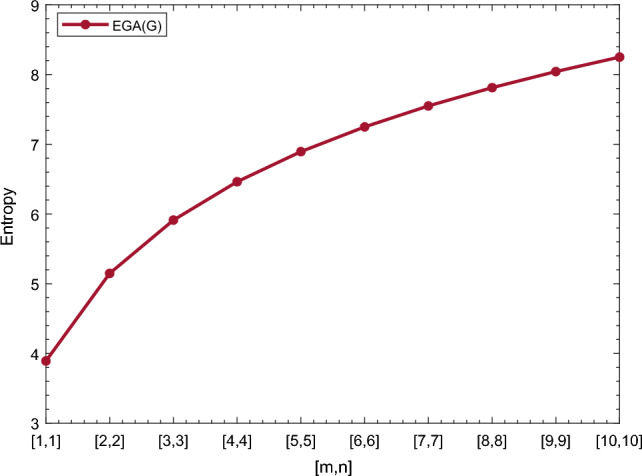
Graphically representation of $E_{GA}$.

**Figure 10:**
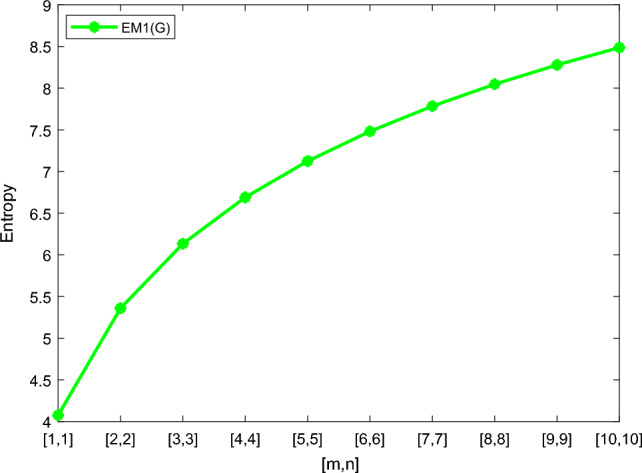
Graphically representation of $E_{M_1}$.

**Figure 11:**
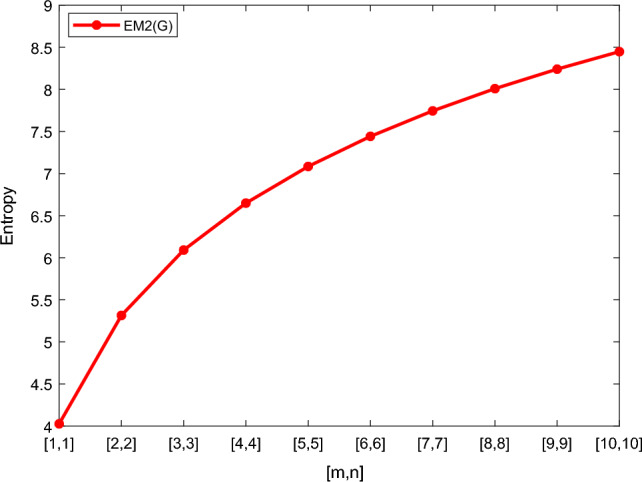
Graphically representation of $E_{M_2}$.

**Figure 12:**
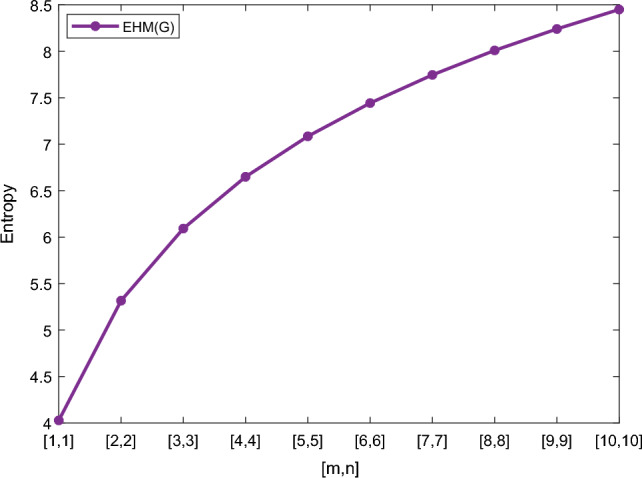
Graphically representation of $E_{HM}$.

**Figure 13:**
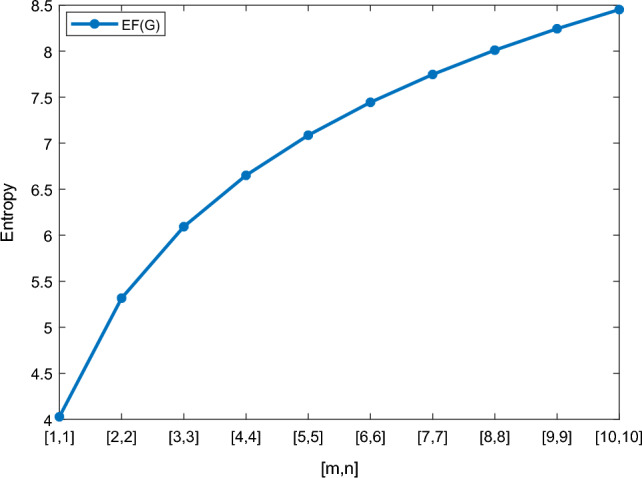
Graphically representation of $E_{F}$.

**Figure 14:**
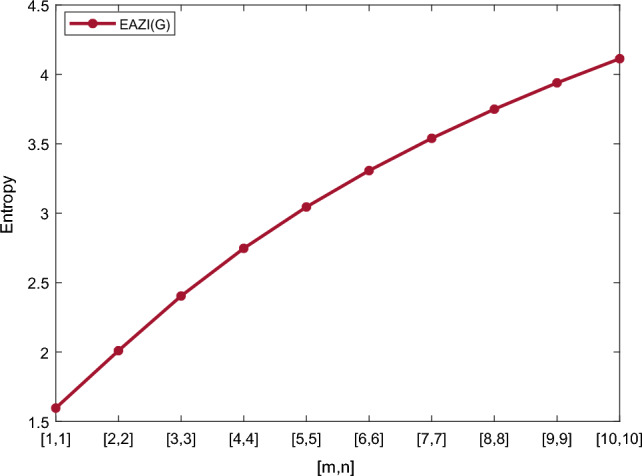
Graphically representation of $E_{AZI}$.

**Figure 15:**
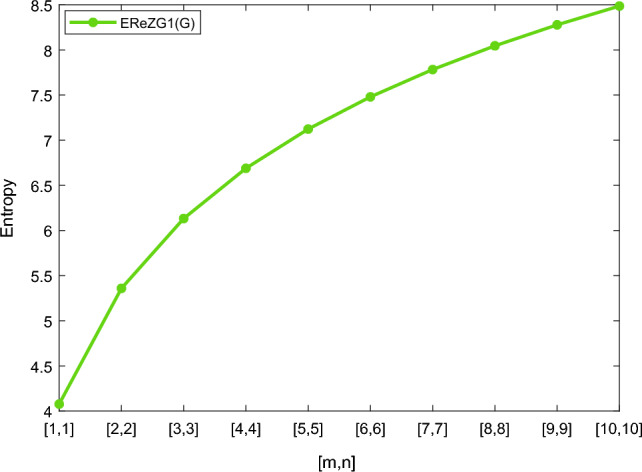
Graphically representation of $E_{ReZG_1}$.

**Figure 16:**
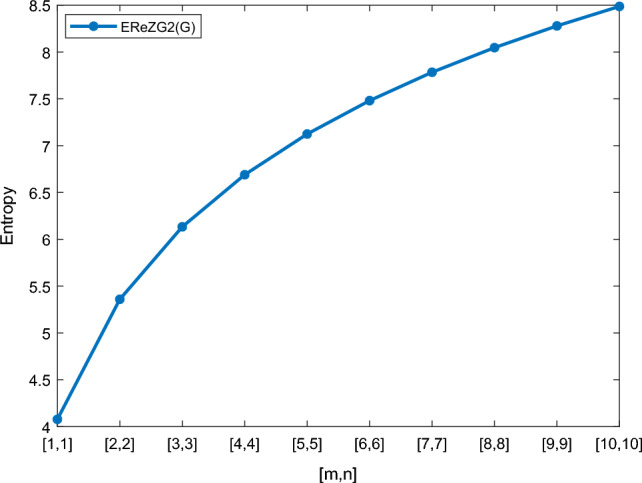
Graphically representation of $E_{ReZG_2}$.

**Figure 17:**
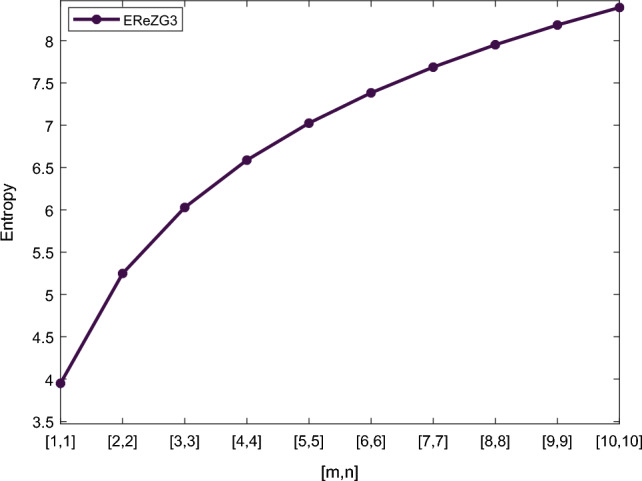
Graphically representation of $E_{ReZG_3}$.

Table 5 shows the numerical results and in Figs. 8, 9, 10 and 11 we represent the graphical comparison of $E_{ABC}$, $E_{GA}$, $E_{M_1}$ and $E_{M_2}$ entropies. The numerical results for $E_{HM}$, $E_{F}$ and $E_{AZI}$ entropies are depicted in Table 6, and graphical comparisons are shown in Figs. 12, 13 and 14 and Table 7 shows the numerical results of redefined Zagreb entropies and graphical comparisons are shown in Figs. 15,16 and 17.

**Table 4:** Numerical comparison of $E_{R_1}$, $E_{R_{-1}}$, $E_{R_{\frac{1}{2}}}$ and $E_{R_{\frac{-1}{2}}}$.

[*m*, *n*]	$E_{R_1}$	$E_{R_{-1}}$	$E_{R_{\frac{1}{2}}}$	$E_{R_{\frac{-1}{2}}}$
[1, 1]	4.0270	4.0287	4.1146	0.94287
[2, 2]	5.3148	5.3110	5.3881	2.1561
[3, 3]	6.0910	6.0856	6.1585	2.9026
[4, 4]	6.6488	6.6425	6.7131	3.4441
[5, 5]	7.0842	7.0776	7.1468	3.8695
[6, 6]	7.4416	7.4347	7.5029	4.2203
[7, 7]	7.7446	7.7377	7.8050	4.5183
[8, 8]	8.0083	8.0008	8.0676	4.7776
[9, 9]	8.2404	8.2330	8.2996	5.0072
[10, 10]	8.4484	8.4413	8.5073	5.2130

**Table 5:** Numerical comparison of $E_{ABC}$, $E_{GA}$, $E_{M_1}$ and $E_{M_2}$.

[*m*, *n*]	$E_{ABC}$	$E_{GA}$	$E_{M_1}$	$E_{M_2}$
[1, 1]	4.0898	3.8932	4.0770	4.0270
[2, 2]	5.3712	5.1491	5.3596	5.3148
[3, 3]	6.1446	5.9125	6.1336	6.0910
[4, 4]	6.7008	6.4632	6.6900	6.6488
[5, 5]	7.1353	6.8944	7.1247	7.0842
[6, 6]	7.4921	7.2489	7.4816	7.4416
[7, 7]	7.7947	7.5498	7.7842	7.7446
[8, 8]	8.0575	7.8114	8.0473	8.0083
[9, 9]	8.2897	8.0426	8.2792	8.2404
[10, 10]	8.4976	8.2498	8.4873	8.4484

**Table 6:** Numerical comparison of $E_{HM}$, $E_{F}$ and $E_{AZI}$.

[*m*, *n*]	$E_{HM}$	$E_{F}$	$E_{AZI}$
[1, 1]	4.0273	4.0274	1.5964
[2, 2]	5.3157	5.3163	2.0096
[3, 3]	6.0922	6.0929	2.4036
[4, 4]	6.6494	6.6507	2.7468
[5, 5]	7.0853	7.0863	3.0450
[6, 6]	7.4428	7.4440	3.3069
[7, 7]	7.7461	7.7466	3.5397
[8, 8]	8.0098	8.0107	3.7494
[9, 9]	8.2406	8.2435	3.9393
[10, 10]	8.4503	8.4519	4.1131

**Table 7:** Numerical comparison of $E_{ReZG_1}$, $E_{ReZG_2}$ and $E_{ReZG_3}$.

[*m*, *n*]	$E_{ReZG_1}$	$E_{ReZG_2}$	$E_{ReZG_3}$
[1, 1]	4.0774	4.0770	3.9505
[2, 2]	5.3590	5.3592	5.2482
[3, 3]	6.1326	6.1332	6.0280
[4, 4]	6.6889	6.6894	6.5874
[5, 5]	7.1234	7.1241	7.0246
[6, 6]	7.4803	7.4808	7.3825
[7, 7]	7.7829	7.7837	7.6863
[8, 8]	8.0457	8.0465	7.9505
[9, 9]	8.2781	8.2788	8.1838
[10, 10]	8.4861	8.4869	8.3908

We have examined degree-based molecular descriptors for the nickel(II) porphyrins Network. The Randic index has a strong correlation with a variety of physicochemical characteristics of alkanes, including chromatographic retention times, surface area, vapor pressure, and boiling temperature variables in the Antonie equation. The atom-bond connectivity (ABC) index proves highly effective in calculating the strain energy of molecules through correlation. A good quantitative structural property relationship (QSPR) model is created when the temperature of alkane production is described by using the ABC index with a high correlation coefficient (r = 0.9970). Moreover, the geometric arithmetic index is a stronger correlation coefficient across a range of physicochemical parameters for octanes. Zagreb indices have been utilized to investigate complexity and hetero systems. These indices are also applied for constructing multilinear regression models and are instrumental in studies related to Quantitative Structure-Property Relationship (QSPR) and Quantitative Structure-Activity Relationship (QSAR)^43,44^. The Forgotten index has demonstrated associations with numerous chemical attributes of molecules. The Augmented Zagreb index proves to be more effective in correlating with the measurement of strain energy in molecules. Additionally, the Balaban index exhibits greater predictive capability compared to the predictive capacity of the Randic index. The above calculation provides numerical representations of the entropy that are calculated using these degree-based topological indices in Tables 4, 5, 6, 7 and graphical representations in Figs. 4, 5, 6, 7, 8, 9, 10, 11, 12,13, 14, 15, 16 and 17. As depicted in Figures, the values of these indices are directly proportional to the values of [*m*, *n*], where [*m*, *n*] is plotted along the x-axes, and the resulting entropy is plotted along the y-axes. These graphs reveal the differences between each entropy for these topological indices for a specific structure. The computational outcomes underscore that the estimates of degree-based indices are significantly influenced by the values of *m* and *n*, or in other words, by the molecular structure.

## Rational curve fitting between heat of formation and entropy of their corresponding indices

In this part, we explain the ideas of Information Entropy and Heat of Formation (Enthalpy) of nickel(II) porphyrins (NiP). The standard molar enthalpy (HoF) of Porphyrins is +629.8 kjmole$^{-1}$. The following is a mathematical formula to determine Heat of Formation (HOF) for various formula units:

$$\begin{aligned} HOF= & {} \frac{Standard\, \,Molar\, \,HOF}{Avogadro's\, \,Number}\times Formula\, \,Units \end{aligned}$$

Based on the characteristics of its chemical graph structure, this could offer us an effective technique to comprehend the molecular structure of Ni(II) porphyrins. The rational built-in function in MATLAB is used to create models between the Heat of Formation and each information entropy because it provides the lowest RMSE value, which indicates the best match. The Numerical Integrity of fit for entropy versus indices of Ni(II) porphyrins is depicted in Table 8. Enthalpy is a property or state function that resembles energy as a result, it has the same dimensions as energy and is measured in joules or ergs.

**Table 8:** Goodness of fit for HoF versus entropy of indices for nickel(II) porphyrins.

Entropie’s	$Fit-type$	*SSE*	$R^2$	*RMSE*
$E_{R_1}(NiP)$	*rat*43	0.013010	1	0.080641
$E_{R_{-1}}(NiP)$	*rat*43	0.022160	1	0.105300
$E_{R_{\frac{1}{2}}}(NiP)$	*rat*43	0.005942	1	0.054510
$E_{R_{\frac{-1}{2}}}(NiP)$	*rat*52	0.004039	1	0.044940
$E_{R_{ABC}}(NiP)$	*rat*32	0.002442	1	0.024710
$E_{R_{GA}}(NiP)$	*rat*43	0.008907	1	0.066730
$E_{R_{M_1}}(NiP)$	*rat*44	0.182200	1	0.426800
$E_{R_{M_2}}(NiP)$	*rat*43	0.029830	1	0.122100
$E_{R_{HM}}(NiP)$	*rat*44	0.761800	1	0.872800
$E_{R_F}(NiP)$	*rat*43	0.066450	1	0.182300
$E_{R_{AZI}}(NiP)$	*rat*42	0.541700	1	0.424900
$E_{R_{ReZG_1}}(NiP)$	*rat*43	0.011340	1	0.075290
$E_{R_{ReZG_2}}(NiP)$	*rat*32	0.001656	1	0.020350
$E_{R_{ReZG_3}}(NiP)$	*rat*32	0.056020	1	0.118300

2 $$\begin{aligned} HoF(R_1)=\frac{p_1(R_1)^4+p_2(R_1)^3+p_3(R_1)^2+p_4(R_1)+p_5}{(R_1)^3 +q_1(R_1)^2+q_2(R_1)+q_3} \end{aligned}$$

**Figure 18:**
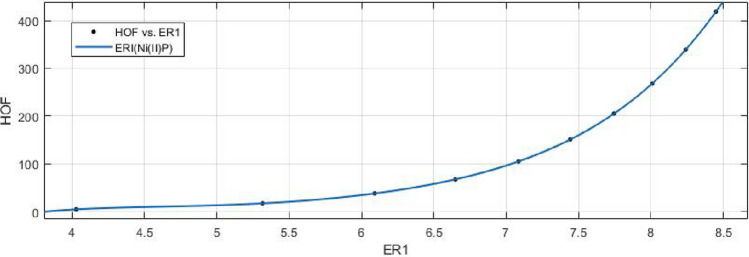
Heat of formation (HoF) versus $E_{R_1}$ of nickel(II) porphyrins.

Where mean 6.9049 and standard deviation 1.4121 are used to normalize the $R_1$ and coefficient (with $95\%$ confidence bounds) are given in Table 9 and graphical representation in Fig. 18.

**Table 9:** Rational curve fitting of HoF versus $E_{R_1}$.

	$p_j$	CI	$q_j$	CI
$j=1$	1293	$(-2.692e+07,2.692e+07)$	480.1	$(-1.087e+07,1.087e+07)$
$j=2$	$-6650$	$(-1.298e+08,1.298e+08)$	$-1.154e+04$	$(-2.524e+08,2.523e+08)$
$j=3$	$-1.648e+04$	$(-4.351e+08,4.351e+08)$	$6.972e+04$	$(-1.507e+09,1.507e+09)$
$j=4$	$2.431e+05$	$(-5.456e+09, 5.456e+09)$		
$j=5$	$-4.891e+05$	$(-1.076e+10, 1.076e+10)$

3 $$\begin{aligned} HoF(R_{-1})= \frac{p_1(R_{-1})^4+p_2(R_{-1})^3+p_3(R_{-1})^2+p_4(R_{-1}) +p_5}{(R_{-1})^3+q_1(R_{-1})^2+q_2(R_{-1})+q_3} \end{aligned}$$

**Figure 19:**
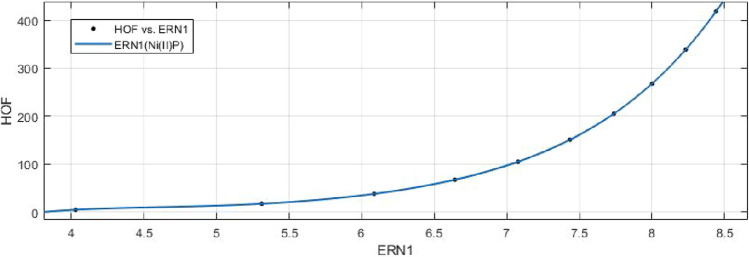
Heat of formation (HoF) versus $E_{R_{\frac{1}{2}}}$ of nickel(II) porphyrins.

Where mean 6.8993 and standard deviation 1.4096 are used to normalize the $R_{-1}$ and coefficient (with $95\%$ confidence bounds) are given in Table 10 graphical representation in Fig. 19.

**Table 10:** Rational curve fitting of HoF versus $E_{R_{-1}}$.

	$p_j$	CI	$q_j$	CI
$j=1$	4677	$(-1.511e+07,1.512e+07)$	$-40.73$	$(-3.703e+04, 3.695e+04)$
$j=2$	$-8.093e+04$	$(-2.616e+08,2.615e+08)$	$-1188$	$(-4.797e+06, 4.795e+06)$
$j=3$	$5.644e+05$	$(-1.824e+09,1.825e+09)$	$1.796e+04$	$(-6.139e+07, 6.143e+07)$
$j=4$	$-1.786e+06$	$(-5.774e+09,5.771e+09)$		
$j=5$	$2.147e+06$	$(-6.934e+09,6.939e+09)$

4 $$\begin{aligned} HoF(R_{\frac{1}{2}})= \frac{p_1(R_{\frac{1}{2}})^4+p_2(R_{\frac{1}{2}})^3+p_3(R_{\frac{1}{2}})^2+p_4(R_{\frac{1}{2}})+p_5}{(R_{\frac{1}{2}})^3+q_1(R_{\frac{1}{2}})^2+q_2(R_{\frac{1}{2}})+q_3} \end{aligned}$$

**Figure 20:**
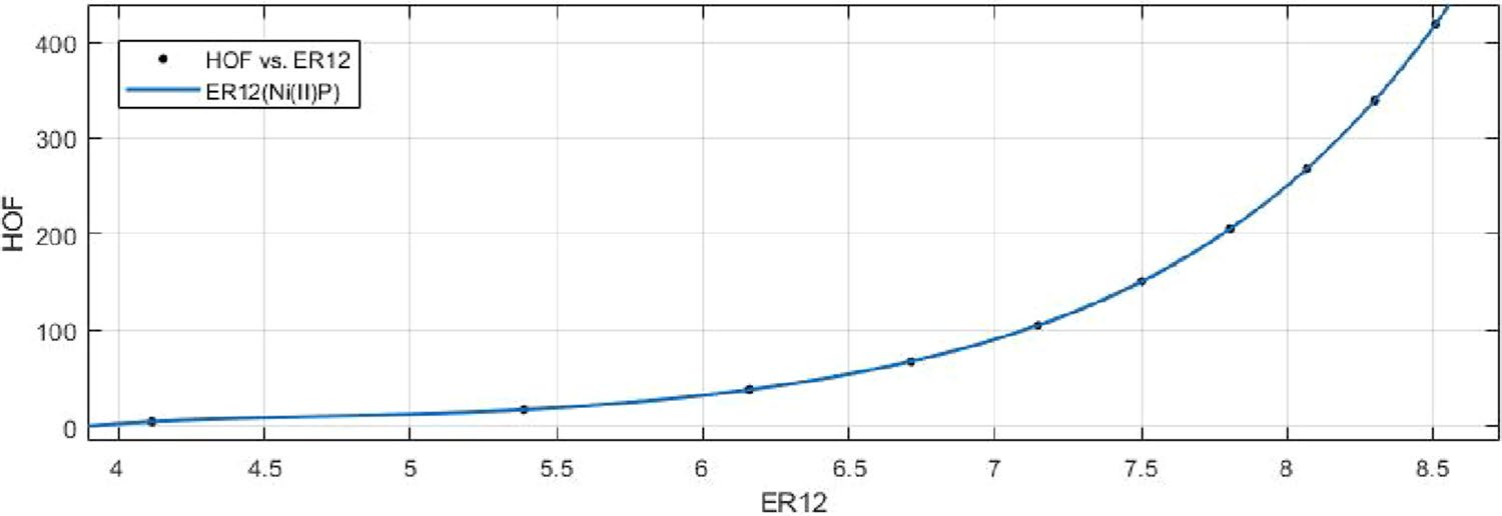
Heat of formation (HoF) versus $E_{R_{\frac{1}{2}}}$ of nickel(II) porphyrins.

Where mean 6.9703 and standard deviation 1.4035 are used to normalize the $R_{\frac{1}{2}}$ and coefficient (with $95\%$ confidence bounds) are given in Table 11 graphical representation in Fig. 20.

**Table 11:** Rational curve fitting of HoF versus $E_{R_{\frac{1}{2}}}$.

	$p_j$	CI	$q_j$	CI
$j=1$	2019	$(-1.081e+07,1.081e+07)$	257.1	$(-1.561e+06, 1.562e+06)$
$j=2$	$-2.605e+04$	$(-1.385e+08,1.385e+08)$	$-6914$	$(-3.923e+07, 3.922e+07)$
$j=3$	$1.453e+05$	$(-7.675e+08,7.678e+08)$	$4.526e+04$	$(-2.514e+08, 2.515e+08)$
$j=4$	$-3.514e+05$	$(-1.838e+09,1.837e+09)$		
$j=5$	$3.11e+05$	$(-1.601e+09,1.601e+09)$

5 $$\begin{aligned} HoF(R_{\frac{-1}{2}})= \frac{p_1(R_{\frac{-1}{2}})^5+p_2(R_{\frac{-1}{2}})^4 +p_3(R_{\frac{-1}{2}})^3+p_4(R_{\frac{-1}{2}})^2+ p_5(R_{\frac{-1}{2}})+p_6}{(R_{\frac{-1}{2}})^2+q_1(R_{\frac{-1}{2}})+q_2} \end{aligned}$$

**Figure 21:**
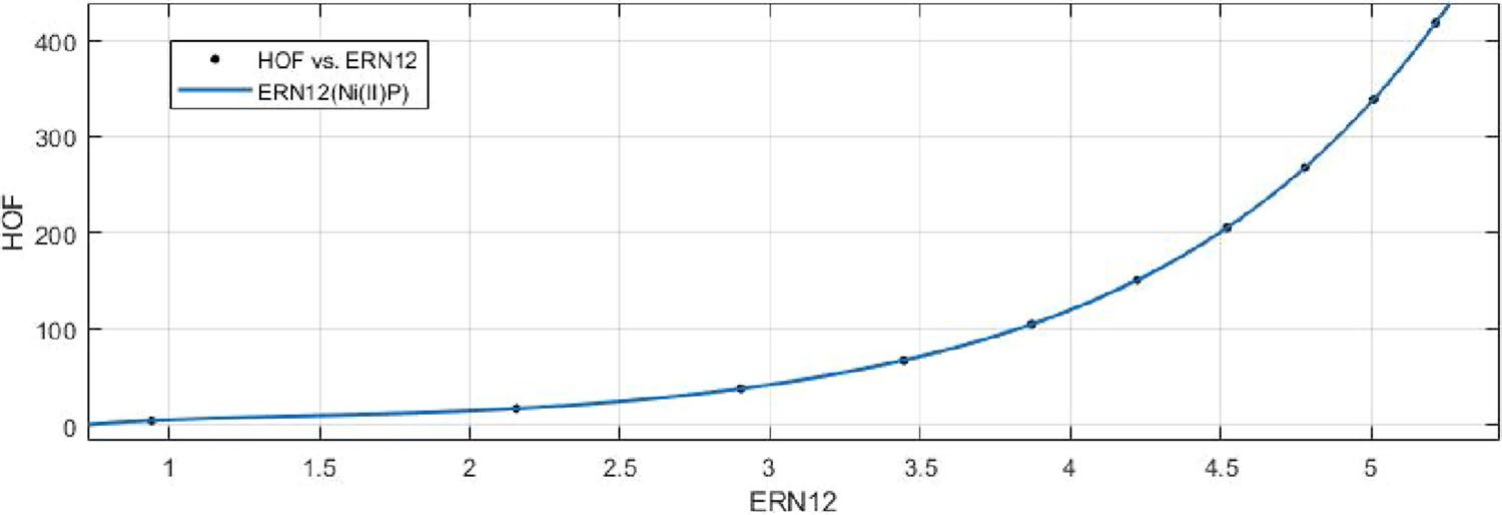
Heat of formation (HoF) versus $E_{R_{\frac{-1}{2}}}$ of nickel(II) porphyrins.

Where mean 3.7052 and standard deviation 1.3669 are used to normalize the $R_{\frac{-1}{2}}$ and coefficient (with $95\%$ confidence bounds) are given in Table 12 and graphical representation in Fig. 21.

**Table 12:** Rational curve fitting of HoF versus $E_{R_{\frac{-1}{2}}}$.

	$p_j$	CI	$q_j$	CI
$j=1$	309.2	$(-3.589e+06, 3.589e+06)$	$-1049$	$(-1.191e+07, 1.191e+07)$
$j=2$	$-422.8$	$(-5.174e+06, 5.174e+06)$	9421	$(-1.079e+08, 1.079e+08)$
$j=3$	2928	$(-3.497e+07, 3.497e+07)$		
$j=4$	$1.186e+04$	$(-1.333e+08, 1.334e+08)$		
$j=5$	6663	$(-7.902e+07, 7.903e+07)$		
$j=6$	$1.592e+04$	$(-1.813e+08, 1.814e+08)$

6 $$\begin{aligned} HoF(ABC)= \frac{p_1(ABC)^3+p_2(ABC)^2+p_3(ABC)+p4}{(ABC)^2+q_1(ABC)+q_2} \end{aligned}$$

**Figure 22:**
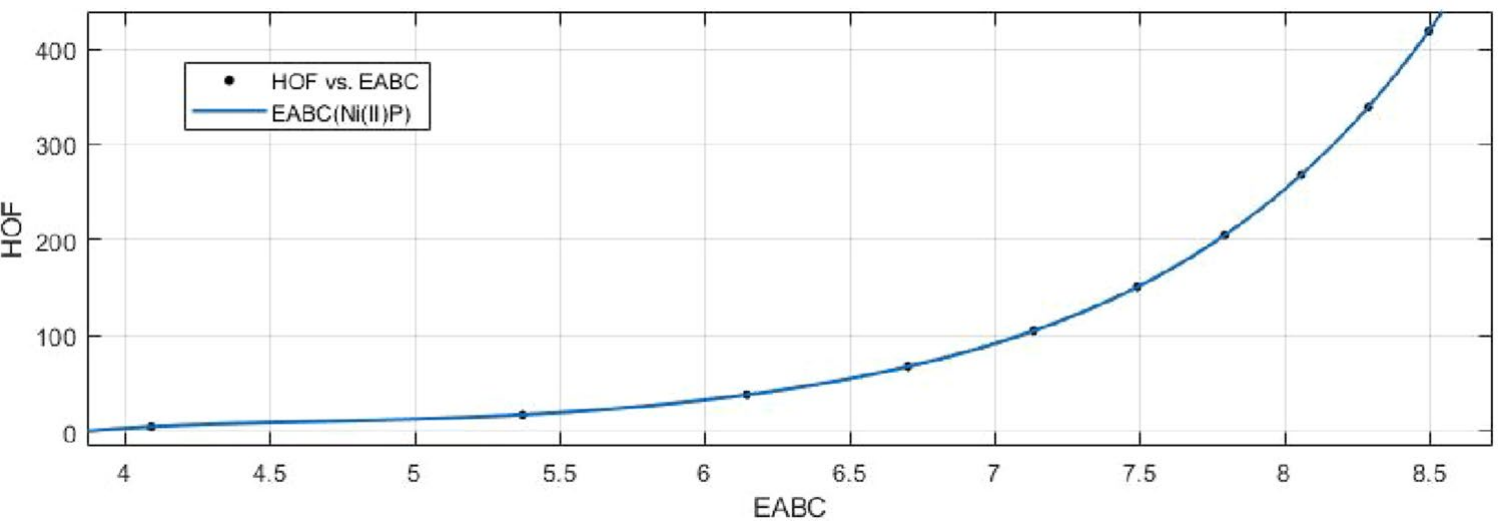
Heat of formation (HoF) versus $E_{ABC}$ of nickel(II) porphyrins.

Where mean 6.9573 and standard deviation 1.4080 are used to normalize the *ABC* and coefficient (with $95\%$ confidence bounds) are given in Table 13 and graphical representation in Fig. 22.

**Table 13:** Rational curve fitting of HoF versus $E_{ABC}$.

	$p_j$	CI	$q_j$	CI
$j=1$	26.46	(20.58, 32.34)	$-22.03$	$(-22.48, -21.57)$
$j=2$	$-283.4$	$(-363.3, -203.6)$	124.9	(119.9, 129.9)
$j=3$	1189	(805.9, 1573)		
$j=4$	$-1718$	$(-2340, -1095)$

7 $$\begin{aligned} HoF(GA)=\frac{p_1(GA)^4+p_2(GA)^3+p_3(GA)^2+p_4(GA)+p_5}{(GA)^3+q_1(GA)^2 +q_2(GA)+q_3} \end{aligned}$$

**Figure 23:**
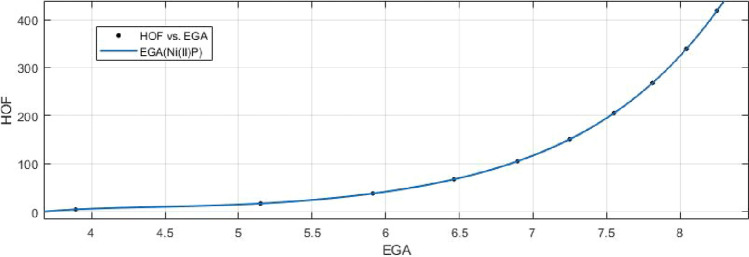
Heat of formation (HoF) versus $E_{GA}$ of nickel(II) porphyrins.

Where mean 6.7215 and standard deviation 1.3927 are used to normalize the *GA* and coefficient (with $95\%$ confidence bounds) are given in Table 14 and graphical representation in Fig. 23.

**Table 14:** Rational curve fitting of HoF versus $E_{GA}$.

	$p_j$	CI	$q_j$	CI
$j=1$	192.1	$(-1.849e+07, 1.849e+07)$	487.7	$(-6.063e+07, 6.063e+07)$
$j=2$	$1.46e+04$	$(-1.784e+09, 1.784e+09)$	$-1.117e+04$	$(-1.344e+09, 1.344e+09)$
$j=3$	$-1.689e+05$	$(-2.03e+10, 2.03e+10)$	$6.447e+04$	$(-7.667e+09, 7.667e+09)$
$j=4$	$7.279e+05$	$(-8.692e+10, 8.693e+10)$		
$j=5$	$-1.061e+06$	$(-1.263e+11, 1.263e+11)$

8 $$\begin{aligned} HoF(M_1)= \frac{p_1(M_1)^4+p_2(M_1)^3+p_3(M_1)^2+p_4(M_1)+p_5}{(M_1)^4+ q_1(M_1)^3+q_2(M_1)^2+q_3(M_1)+q_4} \end{aligned}$$

**Figure 24:**
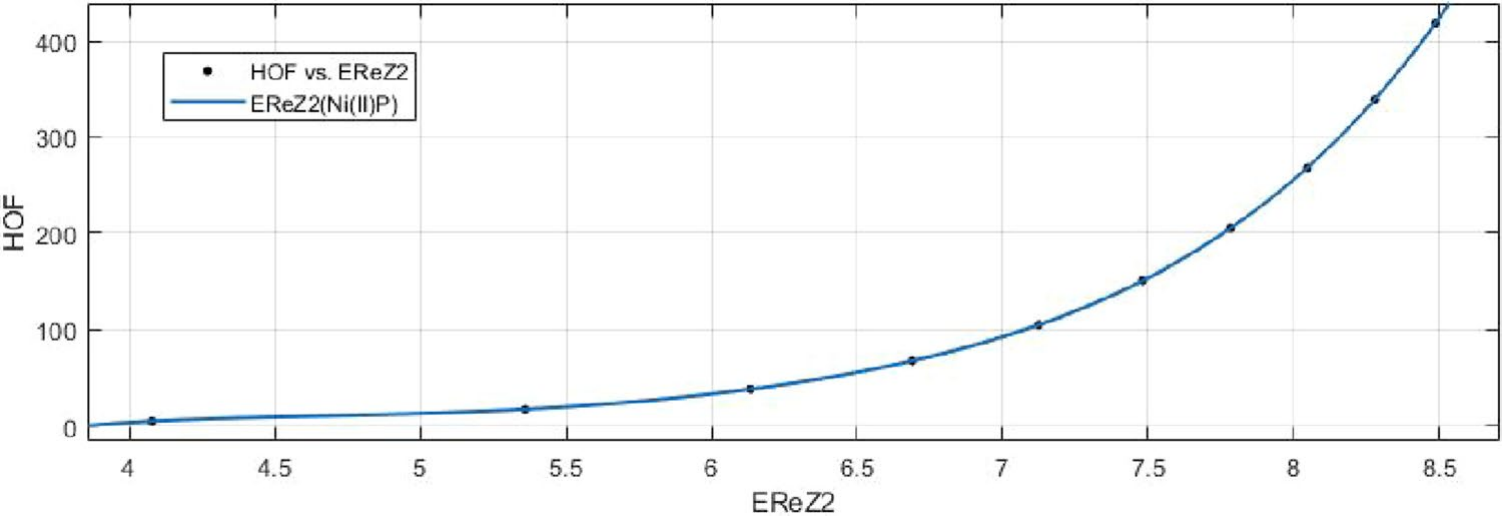
Heat of formation (HoF) versus $E_{M_1}$ of nickel(II) porphyrins.

Where mean 6.9465 and standard deviation 1.4087 are used to normalize the $M_1$ and coefficient (with $95\%$ confidence bounds) and given in Table 15 and graphical representation in Fig. 24.

**Table 15:** Rational curve fitting of HoF versus $E_{M_1}$.

	$p_j$	CI	$q_j$	CI
$j=1$	101.1	$(-4.217e+06, 4.217e+06)$	$-17.34$	$(-4.485e+08, 4.485e+08)$
$j=2$	$-641.6$	$(-4.549e+10, 4.549e+10)$	48.03	$(-7.937e+09, 7.937e+09)$
$j=3$	1071	$(-3.065e+11, 3.065e+11)$	275.3	$(-2.424e+10, 2.424e+10)$
$j=4$	595.4	$(-5.978e+11, 5.978e+11)$	128.1	$(-1.177e+11, 1.177e+11)$
$j=5$	203.7	$(-6.494e+10, 6.494e+10)$

9 $$\begin{aligned} HoF(M_2)= \frac{p_1(M_2)^4+p_2(M_2)^3+p_3(M_2)^2+p_4(M_2)+p_5}{(M_2)^3+ q_1(M_2)^2+q_2(M_2)+q_3} \end{aligned}$$

**Figure 25:**
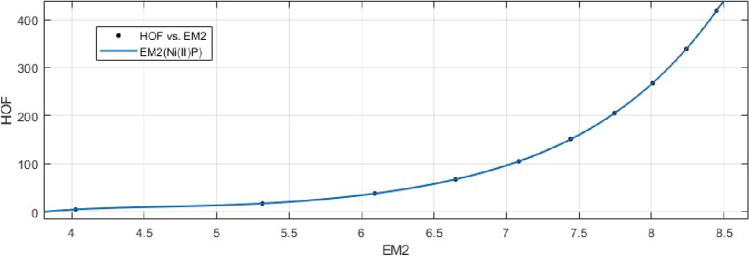
Heat of formation (HoF) versus $E_{M_2}$ of nickel(II) porphyrins.

Where mean 6.9049 and standard deviation 1.4121 are used to normalize the $M_2$ and coefficient (with $95\%$ confidence bounds) are given in Table 16 and graphical representation in Fig. 25.

**Table 16:** Rational curve fitting of HoF versus $E_{M_2}$.

	$p_j$	CI	$q_j$	CI
$j=1$	$-106.8$	$(-5.435e+05, 5.433e+05)$	67.49	$(-4.125e+05, 4.127e+05)$
$j=2$	3899	$(-1.814e+07, 1.814e+07)$	$-1761$	$(-8.728e+06, 8.724e+06)$
$j=3$	$-3.296e+04$	$(-1.502e+08, 1.501e+08)$	$1.018e+04$	$(-4.735e+07, 4.737e+07)$
$j=4$	$1.187e+05$	$(-5.35e+08, 5.352e+08)$		
$j=5$	$-1.523e+05$	$(-6.813e+08, 6.81e+08)$

10 $$\begin{aligned} HoF(HM)= \frac{p_1(HM)^4+p_2(HM)^3+p_3(HM)^2+p_4(HM)+p_5}{(HM)^4 +q_1(HM)^3+q_2(HM)^2+q_3(HM)+q_4} \end{aligned}$$

**Figure 26:**
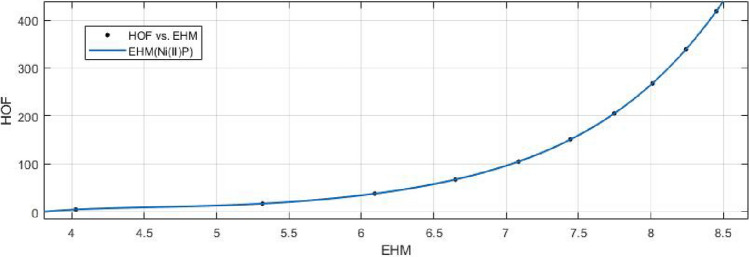
Heat of formation (HoF) versus $E_{HM}$ of nickel(II) porphyrins.

Where mean 6.9059 and standard deviation 1.4124 are used to normalize the *HM* and coefficient (with $95\%$ confidence bounds) are given in Table 17 and graphical representation in Fig. 26.

**Table 17:** Rational curve fitting of HoF versus $E_{HM}$.

	$p_j$	CI	$q_j$	CI
$j=1$	66.7	$(-5.88e+06, 5.88e+06)$	$-16.66$	$(-1.612e+09, 1.612e+09)$
$j=2$	$-329.8$	$(-1.078e+11, 1.078e+11)$	42.03	$(-2.725e+10, 2.725e+10)$
$j=3$	409.8	$(-5.609e+11, 5.609e+11)$	269.6	$(-7.381e+10, 7.381e+10)$
$j=4$	273.4	$(-7.972e+11, 7.972e+11)$	88.98	$(-4.256e+11, 4.256e+11)$
$j=5$	110.6	$(-2.952e+11, 2.952e+11)$

11 $$\begin{aligned} HoF(F)= \frac{p_1(F)^4+p_2(F)^3+p_3(F)^2+p_4(F)+p_5}{(F)^3+q_1(F)^2+ q_2(F)+q_3} \end{aligned}$$

**Figure 27:**
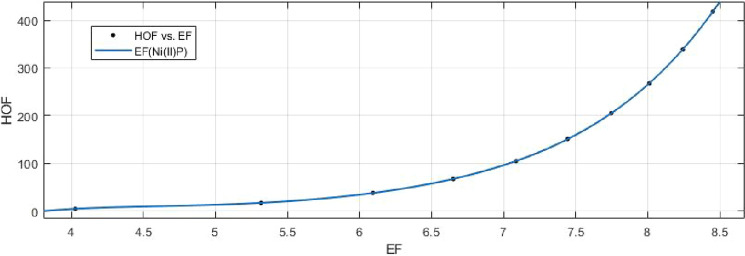
Heat of formation (HoF) versus $E_{F}$ of nickel(II) porphyrins.

Where mean 6.9070 and standard deviation 1.4129 are used to normalize the *F* and coefficient (with $95\%$ confidence bounds) are given in Table 18 and graphical representation in Fig. 27.

**Table 18:** Rational curve fitting of HoF versus $E_{F}$.

	$p_j$	CI	$q_j$	CI
$j=1$	289	$(-1.608e+06, 1.609e+06)$	81.54	$(-6.42e+05, 6.421e+05)$
$j=2$	$-3816$	$(-2.111e+07, 2.11e+07)$	$-2118$	$(-1.394e+07, 1.394e+07)$
$j=3$	$2.759e+04$	$(-1.549e+08, 1.549e+08)$	$1.259e+04$	$(-7.84e+07, 7.843e+07)$
$j=4$	$-9.57e+04$	$(-5.441e+08, 5.439e+08)$		
$j=5$	$1.343e+05$	$(-7.729e+08, 7.731e+08)$

12 $$\begin{aligned} HoF(AZI)= \frac{p_1(AZI)^4+p_2(AZI)^3+p_3(AZI)^2+p_4(AZI)+p_5}{(AZI)^2 +q_1(AZI)+q_2} \end{aligned}$$

**Figure 28:**
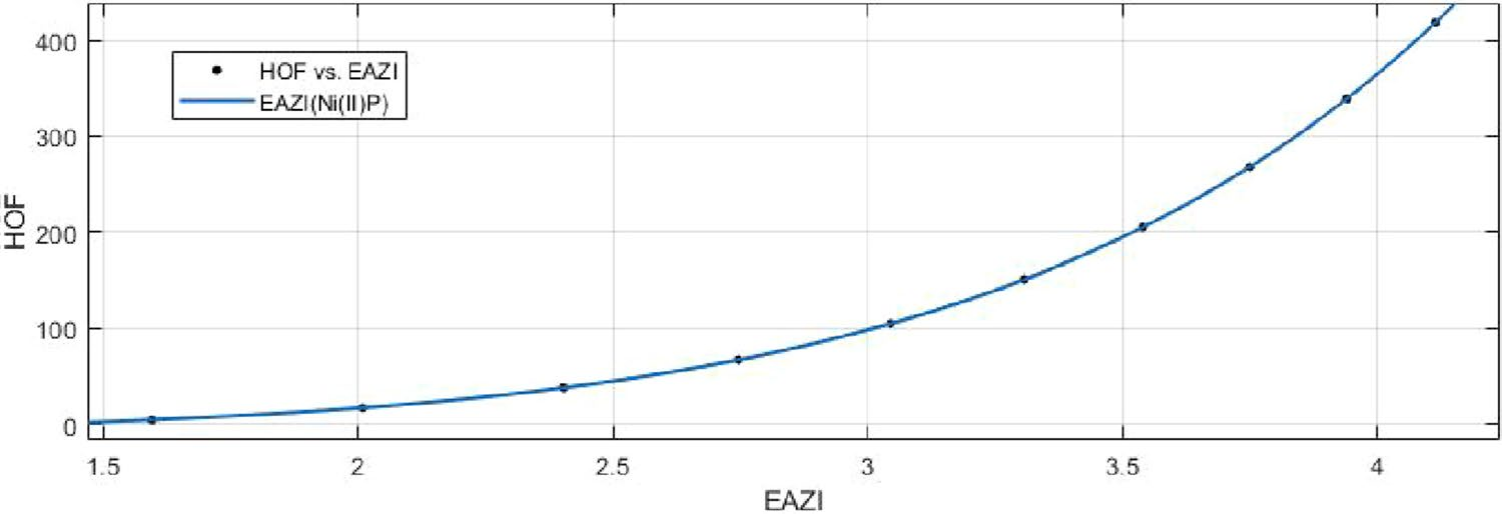
Heat of formation (HoF) versus $E_{AZI}$ of nickel(II) porphyrins.

Where mean 3.0450 and standard deviation 0.8447 are used to normalize the *AZI* and coefficient (with $95\%$ confidence bounds) are given in Table 19 and graphical representation in Fig. 28.

**Table 19:** Rational curve fitting of HoF versus $E_{AZI}$.

	$p_j$	CI	$q_j$	CI
$j=1$	1152	$(-3.346e+08, 3.346e+08)$	$-2154$	$(-6.123e+08, 6.122e+08)$
$j=2$	$3.439e+04$	$(-9.792e+09, 9.792e+09)$	$1.432e+04$	$(-4.081e+09, 4.081e+09)$
$j=3$	$-2.185e+04$	$(-6.211e+09, 6.211e+09)$		
$j=4$	$-8269$	$(-2.385e+09, 2.385e+09)$		
$j=5$	$-2.839e+04$	$(-8.082e+09, 8.082e+09)$

13 $$\begin{aligned} HoF(ReZG_1)=\frac{p_1(ReZG_1)^3+p_2(ReZG_1)^2+p_3(ReZG_1)+p_4}{(ReZG_1)^2+q_1(ReZG_1)+q_2} \end{aligned}$$

**Figure 29:**
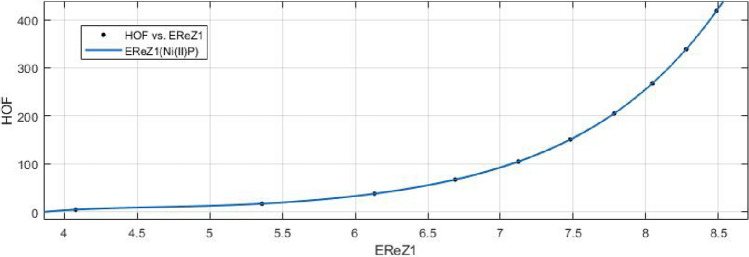
Heat of formation (HoF) versus $E_{ReZG_1}$ of nickel(II) porphyrins.

Where mean 6.9454 and standard deviation 1.4082 are used to normalize the $ReZG_1$ and coefficient (with $95\%$ confidence bounds) are given in Table 20 and graphical representation in Fig. 29.

**Table 20:** Rational curve fitting of HoF versus $E_{ReZG_1}$.

	$p_j$	CI	$q_j$	CI
$j=1$	48.71	$(-5.667, 103.1)$	$-23.97$	$(-28.56, -19.37)$
$j=2$	$-554.8$	$(-1260, 150.7)$	145.7	(95.87, 195.5)
$j=3$	2390	$(-865.4, 5646)$		
$j=4$	$-3511$	$(-8591, 1570)$		
$$j=5$$

14 $$\begin{aligned} HoF(ReZG_2)=\frac{p_1(ReZG_2)^3+p_2(ReZG_2)^2+p_3(ReZG_2) +p_4}{(ReZG_2)^2+q_1(ReZG_2)+q_2} \end{aligned}$$

**Figure 30:**
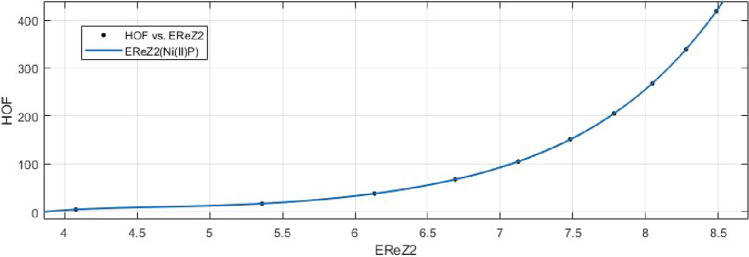
Heat of formation (HoF) versus $E_{ReZG_2}$ of nickel(II) porphyrins.

Where mean 6.9460 and standard deviation 1.4086 are used to normalize the $ReZG_2$ and coefficient (with $95\%$ confidence bounds) are given in Table 21 and graphical representation in Fig. 30.

**Table 21:** Rational curve fitting of HoF versus $E_{ReZG_2}$.

	$p_j$	CI	$q_j$	CI
$j=1$	25.98	(21.24, 30.73)	$-21.95$	$(-22.31, -21.58)$
$j=2$	$-276.5$	$(-340.8, -212.1)$	124.1	(120.1, 128.1)
$j=3$	1155	(847, 1463)		
$j=4$	$-1661$	$(-2161, -1162)$

15 $$\begin{aligned} HoF(ReZG_3)=\frac{p_1(ReZG_3)^3+p_2(ReZG_3)^2+p_3(ReZG_3)+p_4}{ (ReZG_3)^2+q_1(ReZG_3)+q_2} \end{aligned}$$

**Figure 31:**
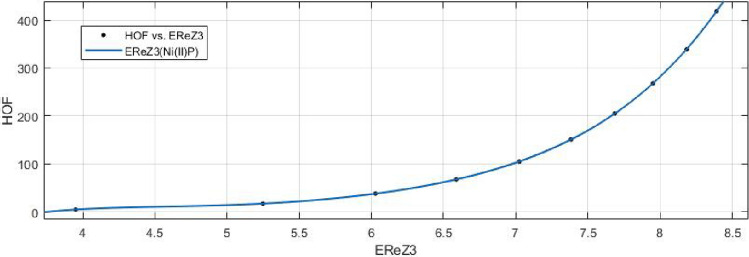
Heat of formation (HoF) versus $E_{ReZG_3}$ of nickel(II) porphyrins.

Where mean 6.8433 and standard deviation 1.4179 are used to normalize the $ReZG_3$ and coefficient (with $95\%$ confidence bounds) are given in Table 22 and graphical representation in Fig. 31.

**Table 22:** Rational curve fitting of HoF versus $E_{ReZG_3}$.

	$p_j$	CI	$q_j$	CI
$j=1$	25.98	(21.24, 30.73)	$-21.95$	$(-22.31, -21.58)$
$j=2$	$-276.5$	$(-340.8, -212.1)$	124.1	(120.1, 128.1)
$j=3$	1155	(847, 1463)		
$j=4$	$-1661$	$(-2161, -1162)$		

Curve fitting of entropy and topological indices can provide insight into the relationship between the structural characteristics of a compound and its thermodynamic and topological properties. This can be useful for predicting the properties of new compounds, understanding the behavior of known compounds, and guiding the design of new materials with desired properties. The heat of formation stands as one of the fundamental physicochemical properties inherent to substances and molecules. We look at the relationship between the heat of formation and the degree-based topological indices (along with their respective entropies) for the nickel(II) porphyrins Network’s corresponding crystal structure. A mathematical connection between the heat of formation and the indices (entropies) is depicted in the data or analysis Tables 8, 9, 10, 11, 12, 13, 14, 15, 16, 17, 18, 19, 20, 21 and 22. The above tables and graphs in the study provide both numerical data and graphical representations that show how the heat of formation is correlated with degree-based topological indices(entropies).

## Conclusion

First, we calculate numerical values for various degree-based topological indices, and next by using the Shanon entropy formula, we calculated entropies by using these indices. After that, we determined the entropy’s graphical behavior using MATLAB and calculated numerical values using Maple. Utilizing MATLAB software, the rational fitting approach was used since it offered the lowest root mean squared error or sum of squared error of all the built-in methods. The findings provide a thorough explanation of the Ni(II) porphyrins crystal structure and demonstrate a strong link between system dimensions and a variety of properties. This research can be used as a theoretical tool to support more effective essential alterations for certain usages by understanding how the characteristics of (NiP) are affected by its shape.

## Data Availability

All data generated or analyzed during this study are included in this published article.
